# Multivariate pattern analysis reveals anatomical connectivity differences between the left and right mesial temporal lobe epilepsy

**DOI:** 10.1016/j.nicl.2014.12.018

**Published:** 2015-01-07

**Authors:** Peng Fang, Jie An, Ling-Li Zeng, Hui Shen, Fanglin Chen, Wensheng Wang, Shijun Qiu, Dewen Hu

**Affiliations:** a College of Mechatronics and Automation, National University of Defense Technology, Changsha, Hunan 410073, China; b Medical Imaging Center, Nanfang Hospital, Southern Medical University, Guangzhou 510515, China; c Medical Imaging Center, Guangdong 999 Brain Hospital, Guangzhou 510510, China

**Keywords:** Temporal lobe epilepsy, Diffusion tensor imaging, Anatomical connectivity, Classification, Cortical-limbic network, Cerebellum

## Abstract

Previous studies have demonstrated differences of clinical signs and functional brain network organizations between the left and right mesial temporal lobe epilepsy (mTLE), but the anatomical connectivity differences underlying functional variance between the left and right mTLE remain uncharacterized. We examined 43 (22 left, 21 right) mTLE patients with hippocampal sclerosis and 39 healthy controls using diffusion tensor imaging. After the whole-brain anatomical networks were constructed for each subject, multivariate pattern analysis was applied to classify the left mTLE from the right mTLE and extract the anatomical connectivity differences between the left and right mTLE patients. The classification results reveal 93.0% accuracy for the left mTLE versus the right mTLE, 93.4% accuracy for the left mTLE versus controls and 90.0% accuracy for the right mTLE versus controls. Compared with the right mTLE, the left mTLE exhibited a different connectivity pattern in the cortical-limbic network and cerebellum. The majority of the most discriminating anatomical connections were located within or across the cortical-limbic network and cerebellum, thereby indicating that these disease-related anatomical network alterations may give rise to a portion of the complex of emotional and memory deficit between the left and right mTLE. Moreover, the orbitofrontal gyrus, cingulate cortex, hippocampus and parahippocampal gyrus, which exhibit high discriminative power in classification, may play critical roles in the pathophysiology of mTLE. The current study demonstrated that anatomical connectivity differences between the left mTLE and the right mTLE may have the potential to serve as a neuroimaging biomarker to guide personalized diagnosis of the left and right mTLE.

## Highlights

• The left mTLE can be identified from the right mTLE with 93.0% accuracy.
• The left mTLE and right mTLE exhibited a different connectivity pattern in cortical-limbic network and cerebellum.
• Anatomical network differences may account for the variance of emotional and memory deficit between the left and right mTLE.

## 1 Introduction

Epilepsy is a chronic brain disorder affecting about 1% of the population worldwide, while mesial temporal lobe epilepsy (mTLE) is the most common type of intractable epilepsy (Almeida et al., 2012)

Recent studies have reported default mode (Liao et al., 2011; McCormick et al., 2013), language (Waites et al., 2006) and sensorimotor network (Voets et al., 2012) disturbances in mTLE, suggesting that mTLE is referred to as a system disorder involving network dysfunctions (Bernhardt et al., 2011; Engel, 2001; Riederer et al., 2008; Waites et al., 2006). The left and the right mTLE are reported to exhibit different clinical performances in emotion, cognition and verbal memory (Hermann et al., 2008). Resting-state fMRI studies showed that the left and right mTLE differed in default mode network (Voets et al., 2012), memory and cognitive network organization (Doucet et al., 2013). Generally, it is reported that left TLE patients have more marked cognitive disorders and impaired executive functions than right TLE patients (Pereira et al., 2010). However, anatomical connectivity, underlying functional variance between the left and right mTLE, is seldom adopted to investigate the direct anatomical differences between the left and right mTLE. As the left and the right mTLE with HS have visually the same brain lesion, only the side matters, the anatomical differences in the left and right mTLE may reveal the variation in neuropathology between them.

Besson et al. demonstrated the anatomical connectivity variance between mTLE and controls, however, no significant connectivity differences were observed in direct univariate statistical comparison of the left mTLE versus the right mTLE (Besson et al., 2014). The difficulty in investigating the direct anatomical connectivity differences between the left and right mTLE is possibly due to the limitations of conventional univariate statistical analysis, which consider the connections independently. Because of the complexity of neuronal networks, the anatomical connectivity differences between the left and right mTLE are encoded by multiple connections which could likely be detected by multivariate pattern analysis (Walther et al., 2009). Multivariate pattern analysis takes inter-regional correlations into account (Shen et al., 2010) and is therefore may have increased sensitivity in extracting stable patterns from neuroimaging data and detecting subtle and spatially distributed differences in the brain (Pereira et al., 2009b); as such, multivariate pattern analysis provides a promising approach for investigating mTLE that is likely to affect networks of the brain (Gong et al., 2011). Compared with group analysis, multivariate pattern analysis is capable of extracting stable structural or functional patterns from neuroimaging data, and can identify potential neuroimaging-based biomarkers to differentiate patients from controls at an individual subject level (Pereira et al., 2009a; Zhu et al., 2008). In fact, there has been increasing interest in multivariate pattern analysis methods to investigate network disturbances in brain-network diseases such as depression (Zeng et al., 2012), schizophrenia (Shen et al., 2010) and Alzheimer's disease (Zhou et al., 2010). Therefore, multivariate pattern analysis should be well suited to explore the direct anatomical connectivity differences between the left and right mTLE.

Clinically speaking, to study the variation of neuropathology between the left and right mTLE and to provide a biomarker for identification of them, it would be valuable to investigate the direct differences between the left and right mTLE from connectivity perspective. In the current study, we therefore adopted multivariate pattern analysis to characterize the direct anatomical network differences between the left and right mTLE.

## 2 Materials and methods

### 2.1 Ethics statement

This study was approved by the Research Ethics Review Board of the Institute of Mental Health of Southern Medical University. Each participant was informed of the details of the project, and written informed consent was obtained from all participants in accord with the standards of the Declaration of Helsinki. We confirmed that all potential participants who declined to participate or otherwise did not participate were eligible for treatment (if applicable) and were not disadvantaged in any way by not participating in this study. We certify that we have participated sufficiently in the work to take public responsibility for the appropriateness of the experimental design and method, and the collection, analysis, and interpretation of the data. We have reviewed the final version of the manuscript and approve it for publication. We certify that this manuscript has not been published in whole or in part nor is it being considered for publication elsewhere. In addition, the authors of this manuscript have no conflicts of interest.

### 2.2 Participants

We enrolled 43 consecutive right-handed patients suffering from unilateral HS and mTLE who received a presurgical evaluation at the Guangdong 999 Brain Hospital. The diagnosis and lateralization of the seizure focus to the left mTLE (*n* = 22) or the right mTLE (*n* = 21) that were determined based on a comprehensive evaluation, including a detailed history, video-EEG telemetry and neuroimaging. An increase in the T2 fluid-attenuated inverted recovery signal in the hippocampus was used as the diagnostic criterion for HS, and the site of HS was concordant with the epileptogenic site in all patients. None of the patients had a mass lesion (including tumor, vascular malformation or malformations of cortical development) or suffered from traumatic brain injury or any psychiatric disorders, but all patients experienced secondary generalized seizures. After MRI acquisition, all patients received anterior temporal lobectomy. Following qualitative histopathological analysis, HS was detected in all patients. So far, there is no seizure recurrence in post-operation patients. Thirty-nine age-, gender- and education-matched right-handed healthy control participants were recruited for this study. All controls were healthy and free of neurological or psychiatric disorders at the time of the study. The demographic and clinical data are presented in Table 1.

### 2.3 Imaging protocol

All participants were scanned using a 1.5 T Philips Intera MR scanner. During scanning, foam pads were used to reduce head motion and scanner noise. Diffusion-weighted images were obtained using a single-shot echo-planar imaging sequence according to the following parameters: repetition time (TR) = 11,000 ms; echo time (TE) = 71.6 ms; field of view (FOV) = 230 × 230 mm; matrix size = 144 × 144; voxel dimensions = 1.6 × 1.6 × 2 mm; slice thickness = 2 mm; 32 non-collinear diffusion directions with a b-value of 800 s/mm^2^ and one additional volume without diffusion weighting (*b* = 0 s/mm^2^); and 73 transverse slices without gaps, covering the entire brain. We also acquired high-resolution 3D brain anatomical images using a T1-weighted MP-RAGE sequence according to the following parameters: TR = 25 ms, TE = 4.6 ms, FOV = 240 × 240 mm, matrix size = 256 × 256, and 140 contiguous axial slices with slice thickness = 1 mm.

### 2.4 DTI data processing

Images obtained in DICOM format were initially converted to ANALYZE format. Subsequently, the diffusion tensor images were corrected for distortions caused by head motion and eddy currents using affine registration in Eddy Current Correction. After completing these preprocesses, the resulting images were brain extracted using the Brain Extraction Tool (Smith, 2002), and a diffusion tensor model was fit to each voxel using DTIFit to generate images of FA and other parameters.

#### 2.4.1 Cortical parcellation

One critical step in network construction was the parcellation of the cortex into regions of interest (ROIs) (Li et al., 2012). Here, we adopted an automatic ROI parcellation method to parcellate the cortex into 116 ROIs, which comprised the nodes in the network (Fang et al., 2012). First, we registered the b0 images to T1-weighted images. Then, we registered the transformed T1-weighted images to the T1-ICBM152 template in MNI space (Andersson et al., 2007). Finally, the resulting transformation matrix was inverted to warp the automated anatomical labeling atlas to the diffusion-MRI native space.

#### 2.4.2 White matter probabilistic tractography

For each DTI set, the Gaussian kernel size was set to 6 for smoothing prior to reconstruction. Then, the local probability distribution of the fiber directions was estimated for each voxel using BedpostX (Behrens et al., 2003b). Here, we selected a computational model that enabled the automatic estimation of two fiber directions within each voxel, which helped to alleviate the fiber-crossing problem and improved the fiber tracking sensitivity in the brain. We adopted ProbtrackX for probabilistic tractography, which tracked fibers between each pair of ROIs by sampling 5000 streamline fibers per voxel using a turning threshold of 60 degrees. All toolboxes in data processing were included in the FMRIB Software Library's Diffusion Toolbox (FSL) (Smith et al., 2004).

#### 2.4.3 Network construction

**Fig. 1:**
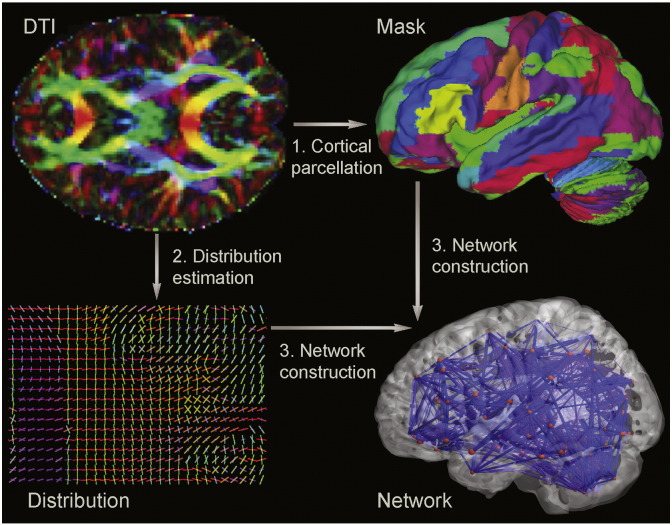
Extraction of a whole-brain anatomical network. The DTI image is presented in a reconstructed color-coded tensor map, showing the directions of the principal axis of diffusion using the standard scheme. Blue codes for the superior–inferior, red for left–right, and green for anterior–posterior orientation. (1) Cortical parcellation. The DTI images are mapped with an AAL atlas in the diffusion-MRI native space. (2) Probability distribution estimation. The computational model enabled the automatic estimation of two fiber directions within each voxel. The color-coding of the estimated fibers is based on a standard RGB code applied to the vector at every segment of each fiber. Blue indicates the rostro-caudal direction; red indicates the medio-lateral plane; and green indicates the dorso-ventral orientation. (3) Whole-brain anatomical network construction. Probabilistic tractography is performed between each pair of ROIs, with only direct connections being retained. Steps 1 and 2 are combined to construct the whole-brain anatomical network.

We combined the output of the cortical parcellation and white matter tractography steps to create an adjacency matrix of brain connectivity. Every ROI in the cortical parcellation became a node in the graph (Sporns, 2011). The ROI associated with node *v* is denoted as *ROI*(*v*). If *ROI*(*v*) contained *n* voxels, the total number of fibers connecting to *ROI*(*v*) was 5000 × *n*. Given the number of fibers from *ROI*(*v*) to *ROI*(*u*) as *m*, the connections between the nodes *ROI*(*v*) and *ROI*(*u*) were defined as edge *e* (*v*, *u*) = $\frac{m}{5000\times n}$. The fibers estimated from *ROI*(*v*) to *ROI*(*u*) did not necessarily match the fibers estimated from *ROI*(*u*) to *ROI*(*v*) because the seed location was randomized in probabilistic tractography. The connectivity strength between *ROI*(*v*) and *ROI*(*u*) was defined as *E* (*v*, *u*) = $\frac{\text{ e (v , u) + e (u , v)}}{2}$. Thus, we obtained a symmetric adjacency matrix of 116 × 116 nodes for each participant (Behrens et al., 2003a). We applied a threshold value of 0.01 to reduce false-positive connections between pairs of ROIs (Gong et al., 2009). Removing the diagonal elements, we selected the upper triangle elements as the classification features. The construction process is displayed graphically in Fig. 1.

### 2.5 Feature selection and classification

#### 2.5.1 Analysis of whole-brain connections

Due to noise, low image resolution, registration error and individual differences, the highly discriminating features, which accounted for only a small portion of the entire feature matrix, were obscured. Thus, our initial step was to select the most discriminating features to construct the feature space for further analysis. We applied a two-sample *t*-test to identify the features that were significantly different between groups. These significantly different features were considered the features that had the most discriminating power. As a manifold learning technique, locally linear embedding (LLE) is capable of obtaining a low-dimensional embedding of the data while preserving the intrinsic data structures (Roweis and Saul, 2000). Therefore, we adopted LLE to reduce the feature space dimensionality to a more manageable level. Finally, a support vector machine (SVM) with the default Gaussian radial basis function kernel was applied for classification (Cortes and Vapnik, 1995). Here, we set up two-way group comparisons in turn for the left mTLE, right mTLE and controls. Additionally, we used a three-way LIBSVM with default parameters to identify the left mTLE, right mTLE and control subjects in a single classification. The LIBSVM adopts a series of one-against-one comparisons to accomplish multi-class classification.

#### 2.3.2 Cross-validation and significance assessment

Because the sample size is limited in this study, we adopted a leave-one-out cross-validation (LOOCV) strategy to estimate the generalization rate (*GR*) of the SVM classifier. The performance of each classifier was quantified for its sensitivity (*SS*), specificity (*SC*) and *GR* based on the results of the LOOCV. The *SS* indicates the proportion of patients that were classified correctly, and the *SC* represents the proportion of controls that were classified correctly. *GR* represents the overall proportion of correctly classified samples. We adopted the same strategy (feature extraction, SVM and LOOCV) for the two-way group comparisons.

To assess the statistical significance of the observed classification accuracy values, we applied permutation tests to evaluate the probability of obtaining *GRs* higher than those obtained using the correct labels by chance. Given the null hypothesis that the observed group differences could have occurred by chance when classifying randomly re-labeled data, we randomly assigned labels to each image and repeated the entire cross-validation procedure 10,000 times (Dosenbach et al., 2010). We counted the number of times that the *GR* for the permuted labels was higher than that obtained using the correct labels. We derived a *p* value for each classification by dividing this number by 10,000.

#### 2.5.3 Analysis with temporal lobe masked out

To assess the direct influence of HS, we repeated the two-way group comparison analyses using connections with temporal lobe masked out. We removed the connections involving temporal lobe ROIs bilaterally and took the remaining connections as features in comparison. The comparison analyses were performed according to the same strategy and parameters as that in the whole-brain classification.

## 3 Results

### 3.1 Whole-brain classification

Using the LOOCV strategy, the SVM classifier achieved 93.0% accuracy for the left mTLE versus the right mTLE, 93.4% accuracy for the left mTLE versus controls and 90.0% accuracy for the right mTLE versus controls. Three-way classification showed a total accuracy of 86.6% (for details, see Table 2).

**Fig. 2:**
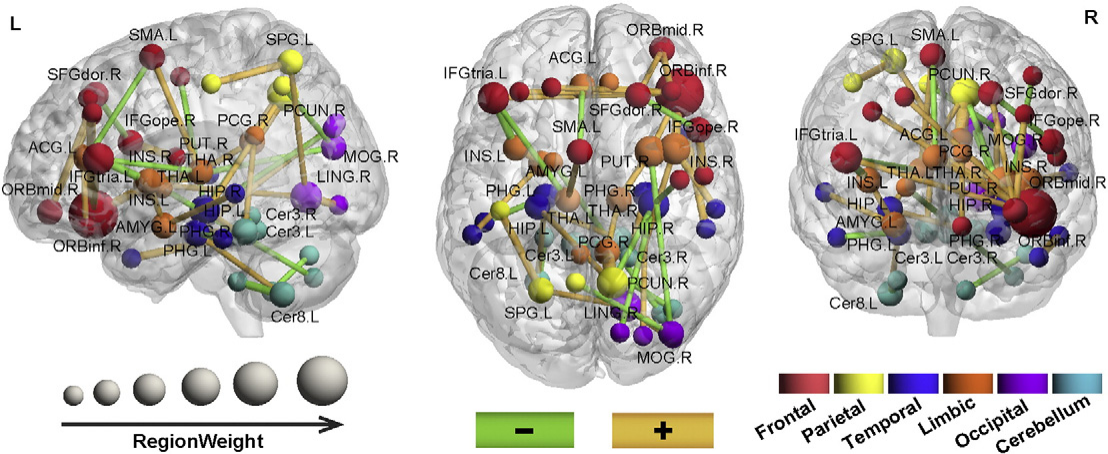
Region weights and distribution of the consensus anatomical connections. The results of classification for the left mTLE versus the right mTLE. The consensus connections are displayed on a surface rendering of the brain. The thickness of the connections adjusts according to their connectivity strength. Green and orange connections represent the decreased and increased connectivity in the left mTLE compared to the right mTLE. The connections are all decreased in the left and right mTLE compared to controls. However, the discriminating connections in the cerebellum and connections with occipital gyrus and ACC were more decreased in the left mTLE compared to those in the right mTLE. The diameter of the sphere represents the corresponding region weight of the ROI. The ROIs are color-coded according to brain areas (red = frontal cortex; orange = limbic cortex; yellow = parietal cortex; sky blue = cerebellar cortex; deep blue = temporal cortex; purple = occipital cortex). R = Right hemisphere, L = left hemisphere. med = medial; mid = middle; ope = opercular; tria = triangular; sup = superior; ORB = orbital frontal; SFG = superior frontal; ACG = anterior cingulum; SMA = supplementary motor area; INS = insula; AMYG = amygdala; PCG = post cingulum; PUT = putamen; HIP = hippocampus; PHG = parahippocampal; TPO = temporal pole; CUN = cuneus; MOG = middle occipital; PCUN = precuneus; LING = lingual; SPG = superior parietal; PoCG = postcentral; Cer = cerebelum.

Because the training data differed for each LOOCV, the selected features varied slightly in each LOOCV. However, 43, 97 and 94 discriminating features, referred to as the consensus features (Dosenbach et al., 2010), were detected in every LOOCV for the left mTLE versus the right mTLE, the left mTLE versus controls and the right mTLE versus controls, respectively. These three sets of consensus features were considered the most discriminating features in the classification. The left mTLE exhibited variant connectivity patterns from the right mTLE in cortical-limbic network and cerebellum (Fig. 2, Table S1). Several ROIs, such as the orbitofrontal gyrus, insula, cingulate cortex, precuneus, hippocampus and parahippocampal gyrus, exhibited high region weights in the classification of the left mTLE versus the right mTLE. The orbitofrontal gyrus showed the greatest discriminative power in the classification and the anatomical connections between the orbitofrontal gyrus and the limbic area, the middle and superior prefrontal cortices were more decreased in the right mTLE than in the left mTLE. Additionally, connections from the hippocampus and parahippocampal gyrus to the cerebellum and occipital cortex may play important role in the neuropathology of mTLE. All the consensus connections were diminished in both the left and the right mTLE compared to the controls (for detail, please see SI, Tables S2 and S3). However, the discriminating connections in cerebellum and connections with occipital gyrus and ACC were more decreased in the left mTLE compared to those in the right mTLE. For visual assessment, the diameter of the sphere was scaled by the corresponding region weight of the ROI (Fig. 2).

### 3.2 Classification using a temporal lobe mask

Classification was also performed on the connections in which the temporal lobe was not involved. The classifications for the left mTLE versus right mTLE, left mTLE versus controls and right mTLE versus controls resulted in accuracies of 90.7%, 90.2% and 88.3%, respectively (Table 2).

## 4 Discussion

In this study, we adopted a probabilistic diffusion tracking method and multivariate pattern analysis to investigate the anatomical connectivity differences between the left and right mTLE. This study demonstrated that the left mTLE can be distinguished from the right mTLE using anatomical connectivity with satisfactory classification accuracy (93.0%) and sensitivity. Moreover, the majority of different connections between the left mTLE and right mTLE were located in cortical-limbic network and cerebellum.

### 4.1 Different anatomical networks between the left and right mTLE

#### 4.1.1 Cortical-limbic network

In direct comparison of the left and right mTLE, the left mTLE showed different connectivity patterns from the right mTLE in cortical-limbic network, involving cortical connections with limbic lobe such as the orbitofrontal gyrus, insula, cingulate cortex, hippocampus and parahippocampal gyrus. The frontal lobe and limbic lobe exhibited the largest region weights in this study. The frontal lobe represents a major route of seizure propagation from the mesial temporal focus, and impaired emotional and cognitive functions of frontal lobe have been reported in TLE (Takaya et al., 2006). Different connections of the orbitofrontal cortex with other prefrontal cortical regions (Cavada et al., 2000) and limbic areas such as insular and putamen (Phillips et al., 2003) (Lothe et al., 2008) may be related to variation in emotion regulation between the left and right mTLE (Cardinal et al., 2002). The ipsilateral hippocampus and parahippocampal gyrus are key components in the generation and propagation of seizure in mTLE subserving memory (Pereira et al., 2010; Sehatpour et al., 2008), and altered connections to the hippocampus and parahippocampal gyrus may be related to deficits in encoding episodic and working memories observed in mTLE (Dörfel et al., 2009; Ranganath, 2010; Squire and Zola-Morgan, 1991). The cingulate cortex, an integral part of the limbic system, is a critical region involved in emotion processing (Hadland et al., 2003) and memory (Kozlovskiy et al., 2012; Kozlovskiy et al., 2013). The anterior cingulate cortex, connecting with the frontal cortex, makes it a central station for processing top-down and bottom-up stimuli and assesses the salience of cognition, emotion and memory (Bush et al., 2000; Kozlovskiy et al., 2013). The posterior cingulate cortex together with precuneus and thalamus are involved in mediation of attention, motivation, emotion and memory (Maddock et al., 2001; Maddock et al., 2003; Pearson et al., 2011). Different connections of the cingulate cortex with the frontal cortex, thalamus and precuneus may account for the different disturbances in emotion and memory between the left and right mTLE. Given this evidence, it was tempting to speculate that the altered cortical-limbic network could lead to different clinical performances in emotion and memory between the left and right mTLE (Doucet et al., 2013).

#### 4.1.2 Cerebellum

Relative to the right mTLE, the left mTLE showed connectivity variation in the cerebellum, including several abnormal connections between the cerebellum and the limbic regions. The posterior cerebellar lobe is primarily involved in cognitive function (Stoodley, 2012) and the atrophy of the posterior cerebellar lobe has been found to be associated with the chronicity of epilepsy (Oyegbile et al., 2011). The vermis has long been labeled the limbic cerebellum subserving the modulation of affective processing and emotion (Bobee et al., 2000; Stoodley and Schmahmann, 2010). In addition, the cerebellum has anatomical connections with the limbic regions, such as the parahippocampal, which are involved in emotion arousal (Zeng et al., 2011). We speculated that the aberrant cerebellar connectivity and their connections with the limbic regions may partially underlie the divergent emotional dysfunction between the left and right mTLE.

### 4.2 Classification

In the present study, based on pair-wise comparisons, the left mTLE was identified from the right mTLE with 93.0% accuracy, 93.4% of the left mTLE were differentiated from the controls and 90.0% of the right mTLE were differentiated from the controls. The results from permutation tests and receiver operating characteristic (ROC) curves, displayed in Figs. S1 and S2, also demonstrated the efficiency of the classifier, suggesting that the most discriminating anatomical connections could serve as biomarkers of mTLE and seizure localization. As the patients in this study experienced secondary generalized seizures, it is hard to identify the left mTLE from the right mTLE from the clinical performances. The classifier distinguished them from each other, indicating that our classifier may be effective while clinical performances are less indicative in seizure localization. To assess the direct influence of HS, we focused on additional classifications using connections with the temporal lobe masked out. The classification achieved 90.7% accuracy for the left mTLE versus the right mTLE, 90.2% accuracy for the left mTLE versus controls and 88.3% accuracy for the right mTLE versus controls, respectively (permutation test and ROC results are displayed in Fig. S1 and Fig. S2). These results suggested that focus showed little impact in classification and our classifier can be used in mTLE with no visual lesion.

Most MRI classification studies focus on two-group comparisons. However, the potential categories are usually not binary in clinical practice but include several distinct possibilities; for instance, classifying the left mTLE versus right mTLE versus controls; major depression versus bipolar depression versus controls and patients versus healthy siblings versus controls. Therefore, it is rather practical to perform three-way classifications for clinical applications. We included the left mTLE, right mTLE and controls for three-way classification in a single analysis. The accuracy for this three-way classification was 86.6% (left mTLE accuracy = 86.4%; right mTLE accuracy = 81.0%; control accuracy = 89.7%). These results confirmed that the left mTLE can be identified from the right mTLE and the three-way classification may have potential for use in future clinical applications.

In this paper, we used the automatic anatomical labeling (AAL) atlas for cortical parcellation. In some studies, Freesurfer brain parcellation (Hagmann et al., 2008), parcellation based on fiber density (Zhang et al., 2010) was used for brain parcellation. However, no brain parcellation is perfect, then our parcellation can be a comparison and complimentary for those studies. As the classification accuracy is quite good, the AAL parcellation method is effective in this study. Some brain parcellation, such as random parcellation (Hagmann et al., 2007) that parcellate brain into thousands of regions may not very suitable for classification. As they parcellate the brain into very small regions, the variability is too big for each region across the whole data set, which may lead the classifier a poor generalization rate for new data. In addition, AAL atlas is the most used and popular atlas for structural connectivity construction (Fenchel et al., 2008; Lawrence et al., 2014; Sandor and Leahy, 1994; Tzourio-Mazoyer et al., 2002; Yan et al., 2011). So, AAL atlas is selected in this study.

### 4.3 Limitations

There are several limitations that should be acknowledged. First, as LOOCV trains training data and predicts test data for N times, it is rather computing consuming. Additionally, LOOCV has high variance (Efron, 1983). In this study, we also applied 10-fold cross-validation strategy for classification, but the result was not as good as the LOOCV, which suggested the bigger the sample size, the better the classification accuracy. The relatively small sample size reduces our confidence in the results. It would be desirable to utilize the method presented in this paper with larger samples. Second, the AAL atlas comprises differently sized anatomical regions that may affect obtained connections. So, in the future, better brain parcellation methods should be adapted to construct the brain network. Third, probabilistic tractography has some intrinsic problems, such as spurious fiber orientations and multi-crossing fibers (Parker and Alexander, 2005); thus, more advanced techniques, such as Diffusion Spectrum Imaging and High Angular Resolution Diffusion Imaging, should be used in the future to confirm our results. Finally, there is no clinical data or EEG data that can be used for correlation analyses in this study. We will collect clinical and EEG data in the future to strengthen our results.

## 5 Conclusion

In conclusion, this study adds a machine learning approach and anatomical network perspective to the current studies of temporal lobe epilepsy. The results demonstrated that the left mTLE can be identified from the right mTLE with 93.0% accuracy. The left mTLE and right mTLE exhibited a different connectivity pattern in cortical-limbic network and cerebellum, indicating that disease-related anatomical network differences may give rise to part of complex variance of emotional and memory deficit between the left and right mTLE. Moreover, the anatomical network differences can be use to differentiate the left mTLE from the right mTLE and anatomical connectivity had its potentiality in prediction and diagnosis of the left and right TLE even with no HS.

## Conflicts of interest

All authors report no disclosures.

## Figures and Tables

**Table 1:** Demographic and clinical data.

Variable	Left mTLE	Right mTLE	Control	*p*-Value
Sample size	22	21	39	
Gender (M/F)	14/8	11/10	22/17	0.45^a^*
Age (median, range in years)	26.2 ± 7.4 (18–42)	28.33 ± 7.8 (18–43)	26.11 ± 7 (18–44)	0.97^a^*
Education (years)	11.1 ± 2	11.5 ± 2.3	11.4 ± 2.28	0.82^a^*
Duration of episode (years)	12.2 ± 7	12.9 ± 7.4		0.74^b^*
Onset of epilepsy (years)	14 ± 9.9	15.6 ± 9.8		0.59^b^*

mTLE = mesial temporal lobe epilepsy; M = male; F = female.

a Pearson's chi-square test.

b Two-sample *t*-test.

* No significant difference between groups (significance defined as *p* < 0.05).

**Table 2:** Comparison of classification performance.

Features	Result			
*GR*	*SS*	*SC*	*PT p*-value
LTLE vs RTLE	93.0%	95.5%	90.5%	<0.0001
LTLE vs controls	93.4%	81.8%	100.0%	<0.0001
RTLE vs controls	90.0%	81.0%	94.9%	<0.0001
LTLE vs RTLE (temporal lobe mask)	90.7%	95.5%	85.7%	<0.0001
LTLE vs controls (temporal lobe mask)	90.2%	81.8%	94.9%	<0.0001
RTLE vs controls (temporal lobe mask)	88.3%	81.0%	92.3%	<0.0001
	LTLE	RTLE	Control	Accuracy
Three way classification	86.4%	81.0%	89.7%	86.6%

*GR* = generalization rate; *SS* = sensitivity; *SC* = specificity; *PT* = permutation test; LTLE /RTLE = left/right mesial temporal lobe epilepsy; vs = versus; ROI = region of interest.
