# Supplementary material for: Multivariate pattern analysis reveals anatomical connectivity differences between the left and right mesial temporal lobe epilepsy
Source: Neuroimage Clin. 2015 Jan 7;7:555–61. doi: 10.1016/j.nicl.2014.12.018 (PMC4375640; doi:10.1016/j.nicl.2014.12.018)
Supplement: Supplementary file 1 — Supplementary Materials. [file mmc1.doc]

**Supplemental materials**

**Methods**

**DTI data processing**

We used automatic parcellation method for region of interest (ROI) segmentation and a standard probabilistic tractography algorithm for fiber tracking. ROI segmentation and fiber tracking were all implemented by FSL (<http://www.fmrib.ox.ac.uk/fsl>) . In contrast with the traditional deterministic-streamline tracking algorithm, the probabilistic algorithm does not simply track WM fibers from voxel to voxel but models local diffusion properties and estimates their directions and probabilities. This algorithm generates posterior distributions on the principal direction of diffusion by Markov Chain Monte Carlo (MCMC) sampling and Bayesian inference .

Extraction of the structural network was implemented in the following manner, which was displayed graphically in Figure 1:

1. Cortical parcellation. The automated anatomical labeling (AAL) atlas was applied to parcellating the entire cerebral cortex into 116 regions (58 in each hemisphere). First, all images were skullstripped using the FSL Brain Extraction Tool (BET) . Then, the skullstripped T1-weighted MP-RAGE images were registered to the skullstripped b0 image using a 12-parameter affine registration with a mutual information cost function implemented in Flirt (FSL tool) and a nonlinear registration implemented with FNIRT (FSL tool) . Finally, the transformed T1-weighted images were registered to the skullstripped T1 template of ICBM152 in Montreal Neurological Institute (MNI) space with Flirt and the transformation matrix resulted from step 2 was inversed to warp the AAL atlas from the MNI space to the diffusion-MRI native space. In this manner, we obtained an AAL template for each subject (Figure 1, step 1).
2. Interregional connectivity based on the probabilistic tractography. The four-dimensional diffusion tensor images were aligned to the first volume with McFlirt (FSL tool) to eliminate head motion error. Then the aligned diffusion tensor images were corrected for distortions due to eddy current using affine registration in Eddy Current Correction (FSL tool). After these preprocesses, a diffusion tensor model was fitted at each voxel using DTIFit (FMRIB Software Library’s Diffusion Toolbox) and followed by estimating the local probability distribution of fiber directions at each voxel with BedpostX (FMRIB Software Library’s Diffusion Toolbox) . Here, a computation model allowing for automatic estimation of two fiber directions within each voxel was selected to improve the tracking sensitivity of nondominant fiber populations in the brain . BedpostX generated the basis for probabilistic tractography using ProbtrackX (FMRIB Software Library’s Diffusion Toolbox) (Figure 1, step 2). The probabilistic tractography was performed between two ROIs with only direct connections by sampling 5000 streamline fibers with a turning threshold of 60 degrees per voxel, and then the probabilistic tractography was further constrained to ignore fibers passing through tissue that had a 50% or an even higher probability of being cerebrospinal fluid or gray matter.

By assuming that the *i*-th ROI contained *n* voxels, and we seeded 5000 samples at each voxel; therefore, the total number of fibers connecting with this ROI was 5000$\times$*n*. Furthermore, if the number of fibers from the *i-*th to the *j-*th ROI was *m*, we obtained the intensity of connectivity from the *i-*th to the *j*-th ROI through dividing 5000$\times$*n* by *m* . The fibers estimated from the *i-*th ROI to the *j-*th ROI did not necessarily match the fibers estimated from *j*-th to *i*-th ROI because seed location affected the probabilistic tractography. The connectivity intensity between two regions was defined by averaging these two intensities, and all the connectivity intensities together constituted the connectivity matrix of a whole brain (Figure 1, step 3). Due to low resolution of the DTI images and limitations of the probabilistic tractography, it was inevitable that there were a few false-positive connections between ROIs. Furthermore, the probability of false-positive connections increased when the estimated connectivity intensity between the two ROIs was relatively low. A threshold value of 0.01 was applied to reducing false-positive connections between ROIs, so as to eliminate the connectivities with extraordinarily low intensities .

**References**

Andersson, J., Jenkinson, M., Smith, S.M., 2007. Non-linear registration, aka Spatial normalisation. FMRIB technical report TR07JA2.

Behrens, T.E., Johansen, B.H., Jbabdi, S., Rushworth, M.F., Woolrich, M.W., 2007. Probabilistic diffusion tractography with multiple fibre orientations: what can we gain? . NeuroImage 34, 144-155.

Behrens, T.E., Johansen, B.H., Woolrich, M.W., Smith, S.M., Wheeler-Kingshott, C.A., Boulby, P.A., Barker, G.J., Sillery, E.L., Sheehan, K., Ciccarelli, O., Thompson, A.J., Brady, J.M., Matthews, P.M., 2003a. Non-invasive mapping of connections between human thalamus and cortex using diffusion imaging. Nat Neuroscience 6, 750-757.

Behrens, T.E., Woolrich, M.W., Jenkinson, M., Johansen, B.H., Nunes, R.G., Clare, S., Matthews, P.M., Brady, J.M., Smith, S.M., 2003b. Characterization and propagation of uncertainty in diffusion-weighted MR imaging. Magn. Reson. Med 50, 1077-1088.

Gong, G., Rosa-Neto, P., Carbonell, F., Chen, Z.J., He, Y., Evans, A.C., 2009. Age- and gender-related differences in the cortical anatomical network. J Neurosci 29(50), 15684-15693.

Jenkinson, M., Bannister, P., Brady, M., Smith, S.M., 2002. Improved optimization for the robust and accurate linear registration and motion correction of brain images. NeuroImage 17(2), 825-841.

Robinson, E.C., Hammers, A., Ericsson, A., Edwards, D., Rueckert, D., 2010. Identifying population differences in whole-brain structural networks: a machine learning approach. NeuroImage 50, 910-919.

Smith, S.M., 2002. Fast robust automated brain extraction. Human Brain Mapping 17(3), 143-155.

Smith, S.M., Jenkinson, M., Woolrich, M.W., Beckmann, C.F., Behrens, T.E., Johansen, B.H., Bannister, P.R., De Luca, M., Drobnjak, I., Flitney, D.E., Niazy, R.K., Saunders, J., Vickers, J., Zhang, Y., De Stefano, N., Brady, J.M., Mathhew, P.M., 2004. Advances in functional and structural MR image analysis and implementation as FSL. NeuroImage 23, 208-219.

Tzourio-Mazoyer, N., Landeau, B., Papathanassiou, D., Crivello, F., Etard, O., Delcroix, N., Mazoyer, B., Joliot, M., 2002. Automated anatomical labeling of activations in SPM using a macroscopic anatomical parcellation of the MNI MRI single-subject brain. NeuroImage 15, 273-289.

**Figure Legends**





[**Figure S1**](#__RefHeading___Toc290824925)**. The permutation distribution of the estimate using a support vector machine (repetition times: 10,000)**. Top (A, B, C): The classifier uses the whole-brain anatomical connections as features. Bottom (D, E, F): The classifier uses connections with temporal lobe masked out. A: Left mTLE versus right mTLE, B: Left mTLE versus controls, C: Right mTLE versus controls, D: Left mTLE versus right mTLE, E: Left mTLE versus controls, F: Right mTLE versus controls. X- and Y-labels represent the generalization rate and occurrence number, respectively. *GR_0_* is the generation rate obtained by the classifier trained on the correct class labels. With the generalization rate as the statistic, this figure reveals that the classifier learned the relationship between the data and the labels with a probability of being incorrect of 0.0001.


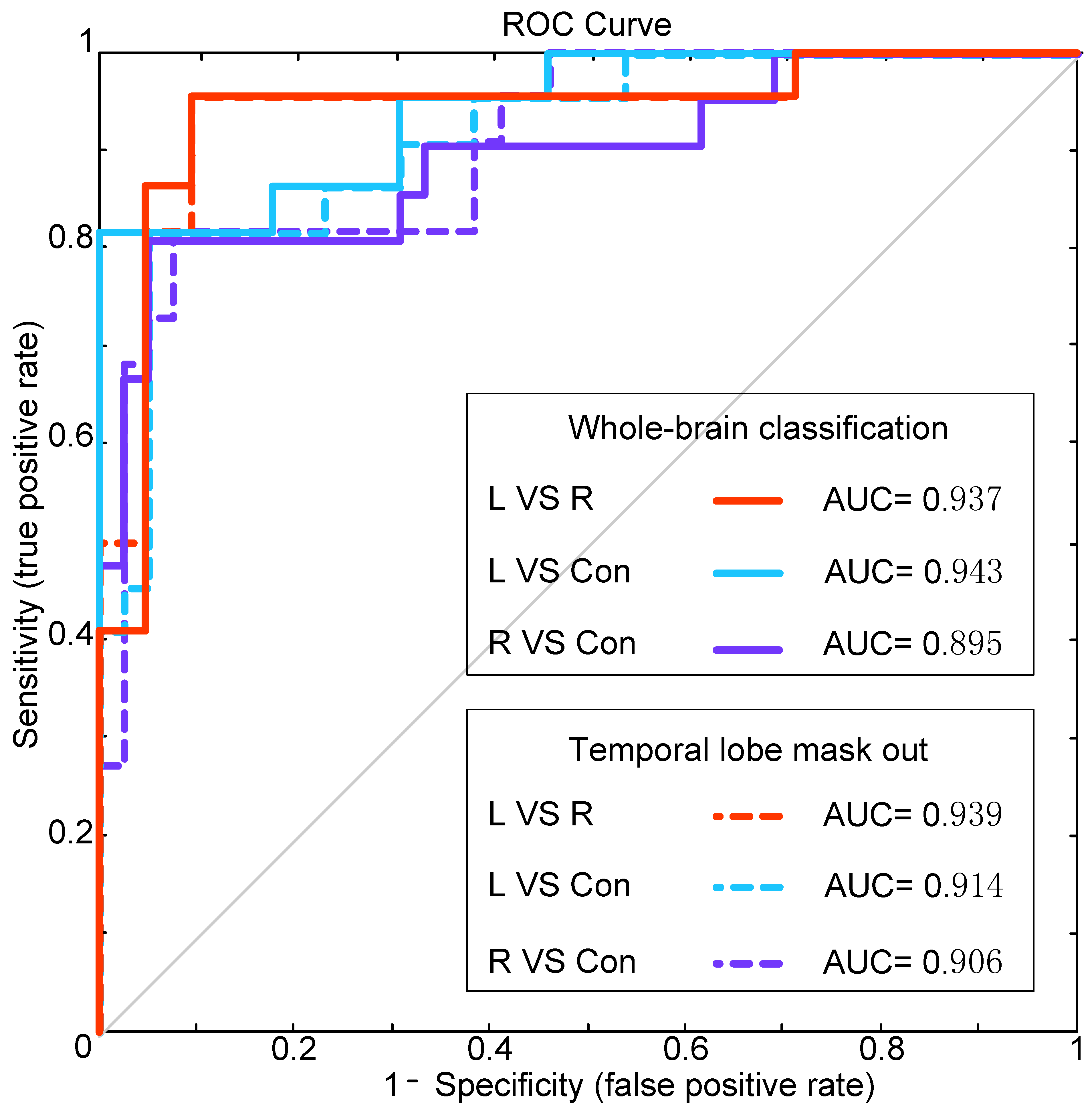


[**Figure S2**](#__RefHeading___Toc290824925)**. ROC curve of the SVM classifiers.** L = left mTLE, R = right mTLE, Con = Control, VS = versus, AUC = area under the curve. Real line and broken line represent ROC curve of the SVM classification with whole-brain connections and with temporal lobe masked out, respectively. Red line corresponds to left mTLE versus right mTLE, sky blue line corresponds to left mTLE versus controls, purple line corresponds to right mTLE versus controls,. This figure indicates that our classifier has satisfactory generalization ability.

**Supplemental Tables**

**Table S1. The consensus features for classification of left TLE versus right TLE (Forty-three consensus anatomical connections between two ROIs). “Network” shows the network to which the connection belongs. L represents the left hemisphere, while R represents the right hemisphere.**

| **Index** | **ROI1** | **ROI2** | **Network** |
| --- | --- | --- | --- |
| 1 | Frontal_Sup_R | Frontal_Inf_Oper_R | Cortical-Limbic network |
| 2 | Frontal_Inf_Oper_R | Rolandic_Oper_R | Cortical-Limbic network |
| 3 | Frontal_Sup_L | Frontal_Inf_Orb_R | Cortical-Limbic network |
| 4 | Frontal_Sup_R | Frontal_Mid_Orb_R | Cortical-Limbic network |
| 5 | Frontal_Mid_L | Frontal_Inf_Orb_R | Cortical-Limbic network |
| 6 | Frontal_Mid_R | Frontal_Mid_Orb_R | Cortical-Limbic network |
| 7 | Frontal_Inf_Tri_L | Frontal_Inf_Orb_R | Cortical-Limbic network |
| 8 | Frontal_Inf_Tri_L | Insula_L | Cortical-Limbic network |
| 9 | Frontal_Inf_Tri_L | Thalamus_L | Cortical-Limbic network |
| 10 | Supp_Motor_Area_L | Cingulum_Ant_L | Cortical-Limbic network |
| 11 | Supp_Motor_Area_L | Thalamus_L | Cortical-Limbic network |
| 12 | Rectus_R | Cingulum_Ant_R | Cortical-Limbic network |
| 13 | Precentral_R | Hippocampus_R | Cortical-Limbic network |
| 14 | Frontal_Inf_Orb_R | Insula_R | Cortical-Limbic network |
| 15 | Frontal_Inf_Orb_R | Cingulum_Ant_L | Cortical-Limbic network |
| 16 | Frontal_Inf_Orb_R | Putamen_R | Cortical-Limbic network |
| 17 | Insula_L | Amygdala_L | Cortical-Limbic network |
| 18 | Cingulum_Post_R | Thalamus_R | Cortical-Limbic network |
| 19 | Putamen_R | Thalamus_R | Cortical-Limbic network |
| **Index** | **ROI1** | **ROI2** | **Network** |
| 20 | Cingulum_Post_R | Precuneus_R | Cortical-Limbic network |
| 21 | Insula_R | Lingual_R | Cortical-Limbic network |
| 22 | Insula_R | Temporal_Sup_R | Cortical-Limbic network |
| 23 | Hippocampus_L | Precuneus_R | Cortical-Limbic network |
| 24 | Hippocampus_R | Precuneus_R | Cortical-Limbic network |
| 25 | Hippocampus_R | Cuneus_R | Cortical-Limbic network |
| 26 | ParaHippocampal_L | Temporal_Inf_L | Cortical-Limbic network |
| 27 | ParaHippocampal_R | Occipital_Sup_R | Cortical-Limbic network |
| 28 | Amygdala_L | Temporal_Mid_L | Cortical-Limbic network |
| 29 | Putamen_R | Occipital_Mid_R | Cortical-Limbic network |
| 30 | Cingulum_Post_L | Cerebelum_3_L | Cerebellum |
| 31 | Hippocampus_L | Cerebelum_3_R | Cerebellum |
| 32 | ParaHippocampal_L | Cerebelum_8_L | Cerebellum |
| 33 | ParaHippocampal_R | Cerebelum_4_5_R | Cerebellum |
| 34 | Pallidum_L | Cerebelum_Crus2_R | Cerebellum |
| 35 | Cerebelum_Crus1_R | Cerebelum_9_R | Cerebellum |
| 36 | Cerebelum_3_L | Vermis_3 | Cerebellum |
| 37 | Cerebelum_3_R | Vermis_1_2 | Cerebellum |
| 38 | Cerebelum_8_L | Cerebelum_10_L | Cerebellum |
| 39 | Lingual_R | Occipital_Inf_R |  |
| 40 | Lingual_R | Parietal_Sup_L |  |
| 41 | Occipital_Mid_R | Precuneus_L |  |
| 42 | Postcentral_L | Parietal_Sup_L |  |
| 43 | Temporal_Pole_Mid_R | Temporal_Inf_R |  |

**Table S2. The consensus features for classification of left TLE versus controls (Ninety-seven consensus anatomical connections between two ROIs).** L represents the left hemisphere, while R represents the right hemisphere.

| **Index** | **ROI1** | **ROI2** |
| --- | --- | --- |
| 1 | Precentral_L | Paracentral_Lobule_L |
| 2 | Precentral_R | Frontal_Sup_R |
| 3 | Precentral_R | Hippocampus_R |
| 4 | Precentral_R | Postcentral_R |
| 5 | Frontal_Sup_L | Frontal_Mid_L |
| 6 | Frontal_Sup_L | Supp_Motor_Area_L |
| 7 | Frontal_Sup_L | Frontal_Sup_Medial_R |
| 8 | Frontal_Sup_L | Cingulum_Ant_L |
| 9 | Frontal_Sup_L | Cingulum_Ant_R |
| 10 | Frontal_Sup_L | Cingulum_Mid_R |
| 11 | Frontal_Sup_R | Frontal_Inf_Oper_R |
| 12 | Frontal_Sup_R | Rolandic_Oper_R |
| 13 | Frontal_Sup_R | Supp_Motor_Area_R |
| 14 | Frontal_Sup_R | Frontal_Med_Orb_R |
| 15 | Frontal_Sup_R | Insula_R |
| 16 | Frontal_Sup_Orb_L | Frontal_Mid_Orb_L |
| 17 | Frontal_Sup_Orb_L | Caudate_R |
| 18 | Frontal_Sup_Orb_R | Frontal_Mid_Orb_R |
| 19 | Frontal_Mid_L | Cingulum_Ant_R |
| 20 | Frontal_Mid_R | Frontal_Sup_Medial_L |
| 21 | Frontal_Mid_Orb_R | Frontal_Inf_Orb_R |
| 22 | Frontal_Inf_Oper_L | Caudate_L |
| 23 | Frontal_Inf_Oper_R | Cingulum_Mid_R |
| 24 | Frontal_Inf_Tri_L | Supp_Motor_Area_R |
| **Index** | **ROI1** | **ROI2** |
| 25 | Frontal_Inf_Tri_L | Insula_L |
| 26 | Frontal_Inf_Tri_L | Caudate_L |
| 27 | Frontal_Inf_Orb_R | Putamen_R |
| 28 | Rolandic_Oper_L | Postcentral_L |
| 29 | Rolandic_Oper_R | Cingulum_Mid_R |
| 30 | Rolandic_Oper_R | Caudate_R |
| 31 | Rolandic_Oper_R | Heschl_R |
| 32 | Supp_Motor_Area_L | Insula_L |
| 33 | Supp_Motor_Area_L | Putamen_L |
| 34 | Supp_Motor_Area_R | Cingulum_Mid_R |
| 35 | Olfactory_L | Frontal_Med_Orb_L |
| 36 | Olfactory_R | Cingulum_Ant_L |
| 37 | Frontal_Sup_Medial_L | Frontal_Sup_Medial_R |
| 38 | Frontal_Sup_Medial_L | Cingulum_Ant_L |
| 39 | Frontal_Sup_Medial_L | Cingulum_Ant_R |
| 40 | Frontal_Med_Orb_L | Frontal_Med_Orb_R |
| 41 | Frontal_Med_Orb_L | Cingulum_Ant_L |
| 42 | Rectus_L | Rectus_R |
| 43 | Insula_L | Caudate_L |
| 44 | Insula_L | Temporal_Pole_Sup_L |
| 45 | Insula_R | Temporal_Pole_Sup_R |
| 46 | Cingulum_Ant_L | Cingulum_Ant_R |
| 47 | Cingulum_Ant_L | Cingulum_Mid_L |
| 48 | Cingulum_Ant_L | Cingulum_Mid_R |
| 49 | Cingulum_Mid_L | Cingulum_Mid_R |
| 50 | Cingulum_Ant_R | Cingulum_Mid_R |
| 51 | Cingulum_Mid_R | Hippocampus_L |
| **Index** | **ROI1** | **ROI2** |
| 52 | Cingulum_Mid_R | Precuneus_R |
| 53 | Cingulum_Post_L | Precuneus_L |
| 54 | Cingulum_Post_L | Temporal_Mid_L |
| 55 | Cingulum_Post_L | Temporal_Inf_L |
| 56 | Cingulum_Post_L | Cerebelum_3_R |
| 57 | Hippocampus_L | Parietal_Sup_L |
| 58 | Hippocampus_L | Precuneus_R |
| 59 | Hippocampus_R | Amygdala_R |
| 60 | Hippocampus_R | Occipital_Inf_L |
| 61 | ParaHippocampal_L | Cerebelum_8_L |
| 62 | Amygdala_L | Amygdala_R |
| 63 | Amygdala_L | Putamen_L |
| 64 | Amygdala_L | Putamen_R |
| 65 | Calcarine_R | Cuneus_R |
| 66 | Calcarine_R | Lingual_R |
| 67 | Cuneus_L | Cuneus_R |
| 68 | Cuneus_L | Temporal_Inf_L |
| 69 | Cuneus_R | Occipital_Sup_R |
| 70 | Cuneus_R | Angular_R |
| 71 | Lingual_R | Temporal_Inf_R |
| 72 | Occipital_Sup_R | Occipital_Mid_R |
| 73 | Occipital_Mid_L | Temporal_Mid_L |
| 74 | Occipital_Mid_R | Temporal_Mid_R |
| 75 | Occipital_Inf_R | Fusiform_R |
| 76 | Fusiform_L | Temporal_Inf_L |
| 77 | Fusiform_R | Temporal_Inf_R |
| 78 | Postcentral_L | SupraMarginal_L |
| **Index** | **ROI1** | **ROI2** |
| 79 | Parietal_Sup_L | Precuneus_L |
| 80 | Parietal_Sup_R | Temporal_Pole_Sup_L |
| 81 | Parietal_Sup_R | Temporal_Mid_L |
| 82 | Parietal_Inf_L | Angular_L |
| 83 | Precuneus_L | Precuneus_R |
| 84 | Precuneus_L | Paracentral_Lobule_R |
| 85 | Precuneus_L | Temporal_Inf_L |
| 86 | Caudate_L | Putamen_L |
| 87 | Putamen_R | Cerebelum_3_L |
| 88 | Pallidum_R | Cerebelum_9_R |
| 89 | Thalamus_L | Cerebelum_3_L |
| 90 | Temporal_Sup_L | Temporal_Mid_L |
| 91 | Temporal_Sup_R | Temporal_Mid_R |
| 92 | Temporal_Pole_Sup_R | Temporal_Mid_R |
| 93 | Temporal_Mid_R | Temporal_Inf_R |
| 94 | Cerebelum_Crus1_R | Cerebelum_10_R |
| 95 | Cerebelum_8_L | Cerebelum_9_R |
| 96 | Cerebelum_8_L | Vermis_4_5 |
| 97 | Cerebelum_9_L | Cerebelum_10_L |

**Table S3. The consensus features for classification of right TLE versus controls (Ninety-two consensus anatomical connections between two ROIs).** L represents the left hemisphere, while R represents the right hemisphere.

| **Index** | **ROI1** | **ROI2** |
| --- | --- | --- |
| 1 | Precentral_L | Postcentral_L |
| 2 | Precentral_R | Frontal_Inf_Oper_R |
| 3 | Frontal_Sup_L | Frontal_Mid_L |
| 4 | Frontal_Sup_R | Frontal_Mid_Orb_R |
| 5 | Frontal_Sup_Orb_L | Frontal_Mid_Orb_L |
| 6 | Frontal_Sup_Orb_R | Frontal_Mid_Orb_R |
| 7 | Frontal_Sup_Orb_R | Rectus_R |
| 8 | Frontal_Mid_L | Cingulum_Ant_R |
| 9 | Frontal_Mid_R | Frontal_Inf_Tri_R |
| 10 | Frontal_Mid_Orb_L | Frontal_Inf_Orb_L |
| 11 | Frontal_Mid_Orb_R | Frontal_Inf_Orb_R |
| 12 | Frontal_Inf_Oper_L | Insula_L |
| 13 | Frontal_Inf_Oper_L | Temporal_Mid_L |
| 14 | Frontal_Inf_Oper_R | Frontal_Inf_Tri_R |
| 15 | Frontal_Inf_Oper_R | Rolandic_Oper_R |
| 16 | Frontal_Inf_Tri_L | Temporal_Pole_Sup_L |
| 17 | Frontal_Inf_Tri_R | Frontal_Inf_Orb_R |
| 18 | Frontal_Inf_Orb_R | Insula_R |
| 19 | Rolandic_Oper_L | Postcentral_L |
| 20 | Rolandic_Oper_R | Insula_R |
| 21 | Rolandic_Oper_R | Postcentral_R |
| 22 | Rolandic_Oper_R | SupraMarginal_R |
| 23 | Rolandic_Oper_R | Heschl_R |
| **Index** | **ROI1** | **ROI2** |
| 22 | Rolandic_Oper_R | Temporal_Sup_R |
| 23 | Supp_Motor_Area_L | Supp_Motor_Area_R |
| 24 | Supp_Motor_Area_L | Frontal_Sup_Medial_L |
| 25 | Olfactory_L | Frontal_Med_Orb_L |
| 26 | Olfactory_L | Frontal_Med_Orb_R |
| 27 | Olfactory_L | Cingulum_Mid_R |
| 28 | Frontal_Sup_Medial_L | Frontal_Sup_Medial_R |
| 29 | Frontal_Sup_Medial_L | Cingulum_Ant_L |
| 30 | Frontal_Sup_Medial_L | Cingulum_Ant_R |
| 31 | Frontal_Med_Orb_L | Frontal_Med_Orb_R |
| 32 | Frontal_Med_Orb_R | Cingulum_Ant_R |
| 33 | Rectus_L | Rectus_R |
| 34 | Rectus_R | Insula_R |
| 35 | Insula_L | Thalamus_L |
| 36 | Insula_L | Temporal_Pole_Sup_L |
| 37 | Insula_R | Putamen_R |
| 38 | Insula_R | Temporal_Sup_R |
| 39 | Cingulum_Mid_L | Cingulum_Mid_R |
| 40 | Cingulum_Mid_L | Precuneus_R |
| 41 | Cingulum_Post_L | Precuneus_L |
| 42 | Cingulum_Post_R | Precuneus_R |
| 43 | Hippocampus_L | Amygdala_L |
| 44 | ParaHippocampal_R | Occipital_Sup_R |
| 45 | ParaHippocampal_R | Fusiform_R |
| 46 | ParaHippocampal_R | Cerebelum_4_5_L |
| 47 | Amygdala_L | Putamen_R |
| 48 | Amygdala_L | Temporal_Mid_L |
| **Index** | **ROI1** | **ROI2** |
| 49 | Calcarine_L | Lingual_L |
| 50 | Calcarine_R | Occipital_Inf_R |
| 51 | Cuneus_L | Precuneus_L |
| 52 | Cuneus_L | Temporal_Inf_L |
| 53 | Cuneus_R | Occipital_Sup_L |
| 54 | Cuneus_R | Occipital_Sup_R |
| 55 | Cuneus_R | Occipital_Mid_L |
| 56 | Cuneus_R | Precuneus_R |
| 57 | Lingual_L | Fusiform_L |
| 58 | Lingual_L | Precuneus_L |
| 59 | Lingual_R | Precuneus_R |
| 60 | Lingual_R | Temporal_Mid_R |
| 61 | Lingual_R | Temporal_Inf_R |
| 62 | Occipital_Sup_L | Occipital_Mid_L |
| 63 | Occipital_Sup_R | Precuneus_R |
| 64 | Occipital_Sup_R | Cerebelum_Crus1_R |
| 65 | Occipital_Mid_L | Parietal_Inf_L |
| 66 | Occipital_Mid_L | Temporal_Sup_L |
| 67 | Occipital_Mid_L | Temporal_Mid_L |
| 68 | Occipital_Mid_R | Precuneus_R |
| 69 | Occipital_Mid_R | Temporal_Inf_L |
| 70 | Occipital_Inf_R | Fusiform_R |
| 71 | Fusiform_L | Temporal_Pole_Mid_L |
| 72 | Fusiform_R | Temporal_Inf_R |
| 73 | Postcentral_L | SupraMarginal_L |
| 74 | Parietal_Inf_L | Paracentral_Lobule_L |
| 75 | Angular_R | Temporal_Sup_L |
| **Index** | **ROI1** | **ROI2** |
| 76 | Precuneus_L | Temporal_Inf_L |
| 77 | Precuneus_R | Caudate_R |
| 78 | Paracentral_Lobule_R | Thalamus_R |
| 79 | Pallidum_L | Cerebelum_6_L |
| 80 | Thalamus_L | Cerebelum_Crus2_L |
| 81 | Heschl_L | Temporal_Sup_L |
| 82 | Heschl_R | Temporal_Sup_R |
| 83 | Temporal_Sup_L | Temporal_Mid_L |
| 84 | Temporal_Sup_R | Temporal_Mid_R |
| 85 | Temporal_Pole_Sup_L | Temporal_Pole_Mid_L |
| 86 | Temporal_Pole_Sup_R | Temporal_Pole_Mid_R |
| 87 | Temporal_Mid_R | Temporal_Pole_Mid_R |
| 88 | Temporal_Mid_R | Temporal_Inf_R |
| 89 | Temporal_Pole_Mid_R | Temporal_Inf_R |
| 90 | Temporal_Inf_L | Cerebelum_7b_L |
| 91 | Cerebelum_Crus1_L | Cerebelum_Crus1_R |
| 92 | Cerebelum_8_L | Cerebelum_10_L |
